# Children’s Caregivers and Public Playgrounds: Potential Reservoirs of Infection of Hand-foot-and-mouth Disease

**DOI:** 10.1038/srep36375

**Published:** 2016-11-07

**Authors:** Pengyuan Li, Tao Li, Qiuyun Gu, Xiaomin Chen, Jiahui Li, Xiashi Chen, Yan Chen, Danwei Zhang, Rong Gao, Zhenjian He, Xun Zhu, Wangjian Zhang, Yuantao Hao, Dingmei Zhang

**Affiliations:** 1 School of Public Health, SunYat-sen University, Guangzhou, China; 2 Medical College of Shaoguan University, Guangdong, China; 3 Zhongshan School of Medicine, SunYat-sen University, Guangzhou, China

## Abstract

Hand-foot-and-mouth disease (HFMD) is a common infectious disease, which has led to millions of clinical cases and hundreds of deaths every year in China. This study aimed to exploring the effects on HFMD transmission of children’s caregivers and public area, as well as trying to locate the potential reservoirs of infections in primary cases. Total children’s 257 samples (98 children’s caregivers and 159 environmental samples) were tested for the presence of universal enterovirus, enterovirus 71, coxsackie virus A6 and A16 by real-time fluorescence quantitative polymerase chain reaction (qPCR). 5.84% (15/257, 95% confidence interval [CI]: 2.98%, 8.70%) of total samples had positive results of enterovirus. The enterovirus positive rates of children’s caregiver samples and environmental samples were respectively 7.14% (7/98, 95% CI: 2.04%, 12.24%), and 5.03% (8/159, 95% CI: 1.63%, 8.43%); 7.61% (7/92, 95% CI: 2.21%, 13.01%) of wiping samples from playgrounds and 1.49% (1/67, 95% CI: 0, 7.00%) of air samples in indoor market places had positive result of enterovirus. High positive rates of enterovirus in children’s caregivers and from playgrounds indicated that they would be potential reservoirs of HFMD infection, as children might be infected via contacting with asymptomatic-infected individuals or exposure of contaminated surface of public facilities.

Hand-foot-mouth disease (HFMD) is one kind of common infectious diseases, which is caused by various enteroviruses from *Picornaviridae* family. Coxsackie A virus and enterovirus 71 (EV71) are the major agents according to the reports data^1^,^2^ while several studies also showed that there are many other types of enterovirus can cause HFMD, such as coxsackievirus A4 (CVA4), coxsackievirus A5 (CVA5), coxsackievirus A10 (CVA10) and coxsackievirus B2 to B5^3^,^4^,^5^. HFMD was named after its major clinical characteristics, and has led to millions of attacks and several outbreaks throughout the world. In the past decade, HFMD has become more predominant in the Asia-Pacific Region and an important issue for global public health^6^,^7^,^8^. In China, there were millions of clinical cases of HFMD and hundreds of deaths per year. 1,997,371 HFMD cases were reported in China during 2015^9^, while 373,198 cases were in Guangdong^10^ (occupied 18.68% of total HFMD cases in China). Guangdong as the most populous province, has been suffering from a large HFMD epidemic.

Transmission of HFMD between human being is via direct contact with respiratory secretions, faeces, herpes fluid or contaminated environment. When HFMD outbreak occurred, kindergartens and schools were the main areas of control and disinfection in mainland of China. Children with HFMD were asked to stay home or hospitals as isolation approach to prevent further spread of disease between children in kindergartens or schools. Day-care nurseries^11^, kindergartens, schools^12^ and rural area^13^ as the main area of HFMD transmission have been well studied. But high level of prevalence of HFMD continuing in China indicates that there might be other reservoirs beside the nurseries, kindergartens and schools. Some reports showed that children had great risk of infection if frequently contacting with infected family members^13^,^14^, or via the virus-existing environment, such as sharing household items^14^, in playgrounds^15^ or shopping malls^16^. In this study, virological investigations were used to explore the effects of children’s caregivers, public playgrounds and air around public area on HFMD transmission, as well as potential reservoirs of infection that could provide evidences for prevention and control of HFMD primary cases.

## Results

### Results of children’s caregivers

Table 1 shows that totally 257 samples were collected and 98 were children’s caregiver samples. From Table 2, 64 participants were between 20 and 39 years old as parents, 26 participants were between 50 and 77 years old as grandparents and eight participants were with unknown age. Totally seven children’s caregiver samples had positive results of enterovirus, of which four people from 20 and 39 years old group and three people from 50 and 77 years old group. Participants with unknown age had no positive result of enterovirus. The enterovirus positive rate of children’s caregiver samples was 7.14% (95% CI: 2.04%, 12.24%). Age group between 20–39 years old had the enterovirus positive rate of 6.25% (4/64) and above 55 years old age group had 11.54% (3/26). There was no statistically significant difference on enterovirus positive rate between two groups (p-value = 0.4074). But none of the samples was detected of positive results of EV71, coxsackievirus A6 (CVA6) or coxsackievirus A16 (CVA16).

### Results of wiping samples in playgrounds

For environmental wiping samples, all 92 samples were collected from ten indoor or outdoor playgrounds in Guangzhou. Seven wiping samples (occupied 7.61% in total wiping samples, 95% CI: 2.21%, 13.01%) were positive for enterovirus. Four out of ten playgrounds were detected positively of enterovirus in Guangzhou. No sample was detected of positive results of EV71, CVA6 or CVA16.

### Results of air samples from public area

67 air samples covered three supermarkets, a bookstore, an indoor playground and a hospital, all with high density of visitors. Only one air sample out of 43 from indoor market places in Shaoguan was detected of enterovirus positively. This air sample was collected in an indoor market place where we arranged eight sample-colleting points totally. Other 24 air samples collected from hospitals and playgrounds had no positive results of enterovirus, and all air samples had no positive detections of EV71, CVA16 or CVA6.

## Discussion

EV71 as one kind of enterovirus, shedding from respiratory tract after onset may continue for nearly four weeks^17^, that is the reason why EV71 can persist in throat secretions for a period of time^18^, and it can survive for a long period outside the host with suitable environment^19^. National Centre for Immunization and Respiratory Diseases (NCIRD) mentioned that some populations, especially adults, might not show any clinical symptoms, but they can still spread the virus to others^20^. Therefore, in this study children’s caregiver samples were collected by nasal and throat swabs and tested for the presence of enterovirus, EV71, CVA16 and CVA6. Overall, seven children’s caregiver samples (7.14%) had been detected positively of enterovirus. Such proportion of children’s caregivers with enterovirus detected involved in this study was higher than in previous reports (enterovirus positive rate of 1.7% on aged above 16-year-old adults from the data of Deng’s study)^21^. In Deng’s study, stool specimens were collected randomly and tested by qPCR. We assume that higher positive rate of enterovirus in our study may due to viruses exist in nasal and throat secretions longer than in stool, although Teng *et al*.^22^ observed that the duration of enterovirus (EV71 and CVA16) last for a long time in patients’ stool. Estimated 7.14% of children’s caregivers have positive enterovirus, means they would be huge reservoir of HFMD viruses that could transmit the viruses to children. In this study we did not collected the nasal and throat swabs from corresponding children of each parent or grandparent simultaneously, the source of infection between children and their caregivers could not be confirmed as there might be a cross propagation occurred between them, but children’s caregivers could be considered as potential reservoir of HFMD infection.

From the results of environmental samples in Table 1, totally seven wiping samples collected from playgrounds (7.61%) had positive results of enterovirus. Playground is one of main transmission places for children and their caregivers. Previous study of Xie had the results that adjusted odds ratios (OR) of hospital HFMD cases to community controls for exposure to public playgrounds were 6.03 (95% CI: 2.84, 12.80), as well as the attributable fractions of this risk factor (57.2%)^15^ was the highest among five risk factors they studied. Such result was also found in reported previous exposures, considered playgrounds as an important risk factor of HFMD^23^. Based on the findings of these previous studies, we collected a large number of wiping samples in public playgrounds in order to estimate the virus level around such kind of area. Just by wiping some points of facilities’ surface, enterovirus rate could be as high as 7.61%. If children continuously wipe or touch the surface more than just some points of it, they will have greater chance of contacting with enterovirus. Our study suggested that enterovirus could be transferred and spread among children by wiping the surface of playground facilities and regular disinfection of surface is essential.

In our study, only one air sample (1.49% of 67 air samples) had positive result. Air samples were collected for 20 minutes long as the minimum collecting time was not well testified, because there was lack of guidelines about air samples of HFMD. Only in sampling plan of swine respiratory pathogens, air samples were collected for detection. Updated methodology used in Corzo’s study^24^ was that collector ran for 30 minutes on air sampling, which was 10 minutes more than the time lasted in our study. Short time period of air samples collection might give rise to the possibilities of virus levels being below the detection limit. If time of air collection increases, more positive results of enterovirus might be detected. As the only one tested positive out of eight samples collected in the same indoor market place, this air sample’s cycle threshold (Ct) value was relatively high (40.93 on average). Such results had not enough evidences to explain air environment of these indoor market places as the reservoir of large number of infectious cases caused by primary virus agents. Besides, high population density of public places might be essential factor to increase the frequency of HFMD transmission through the air, which should be considered carefully in the further studies.

Neither children’s caregiver samples nor environmental samples had been detected positively of predominant virus such as EV71, CVA6 or CVA16^25^. However, other coxsackievirus, such as CVA4, CVA5, CVA10 and CVB2 to B5 might be potential agents of HFMD as well^3^,^4^,^5^. In 2014 in Guangzhou Xintang area, enterovirus was the major pathogen of HFMD among diagnosed cases of HFMD (enterovirus positive rate of 55.76%, CVA16 positive rate of 20.73% and EV71 positive rate of 23.51%)^26^. From this study, other species of enterovirus beside CVA16 and EV71 could be the pathogens of HFMD. Positive detection of enterovirus in our study means the possibility of EV71, CVA6, CVA16 or other coxsackievirus still existing in nasal secretions, throat secretions or surface of facilities in playgrounds, which cannot deny that there is risk of HFMD virus transmission from children’s caregivers or playgrounds. And the reason why no positive detection of EV71, CVA6 or CVA16 could be small sample size that should be increased in further studies.

If children’s caregivers and public playgrounds can serve as the reservoir of the agent of HFMD, it is crucial to the control of HFMD spread. The range of activities for adults is generally far greater than five-years-old children, and there is a greater chance that these caregivers are able to contact with enterovirus. Considering the infection of adults is mostly asymptomatic infection^27^, it makes them difficult to be identified, as preventing and controlling the spread of enterovirus-related diseases. The difficulty can also apply to the public playgrounds. Playgrounds are often fully packed, even during weekdays and especially during the weekends and the holidays. Thus, it would be a challenge to ensure the surface of these recreational facilities pathogen-free and identify the contaminated surface. Overall, 4 out of 10 playgrounds in Guangzhou were detected EV positive as the ratio was 40%. The question remained was whether this result relevant to disinfection measures, visitors’ average daily amount, indoor or outdoor as well as the types of entertainment facilities in these playgrounds.

In order to implement the measurements to prevent and control HFMD among children, disinfection management on public entertainment facilities should be improved. Currently the automatic alert and response system were confirmed that they had good sensitivity on the detection of HFMD infection during the outbreaks^28^, but it is not enough for prevention. Sampling from environment with potential infections and monitoring on general population to identify recessive infection as regular basis should be into the HFMD surveillance system. Future study should be carried on with sampling from children at the same places as their caregivers and tracing back to the HFMD individual cases, because is essential to establish a cohort research and identify the source of HFMD transmission between children and their caregivers. Considering of children’s caregivers, target samples should locate on specific groups such as household members, nurses or tutors. Larger sample size under the calculation of 5% statistical power, and focus on associations between each specific facility and HFMD cases around the same public area, such as children slide and parallel bars. Besides, in this study, enterovirus was detected positively by qPCR, but whether it has infectivity and the infectivity level need cell culture experiments and animal trials to be confirmed.

## Methods

### Ethical issue

All researches involving human participants were approved by the Institutional Review Board of School of Public Health, at the SunYat-sen University, in accordance with the guidelines for the protection of human subjects. Participants provided written informed consent after being briefed on the purpose of the study and of their right to keep information confidential. Written consent was obtained from all study participants.

### Study design

Based on the information and data from samples collection from April 7^th^ to August 4^th^, 2015, laboratory analyses, including extracting RNA, reverse transcription and real-time fluorescence quantitative polymerase chain reaction (qPCR), were used to analyse the positive rates and types of enterovirus within these specimens.

### Samples collection

Total 257 samples were collected in Guangzhou and Shaoguan city, of which 98 nasal and throat swabs were collected from healthy children’s caregivers who were the guardians without any HFMD symptoms and at least have one child aged under five-year-old in community or playgrounds in Guangzhou.

159 samples were from environment, of which 67 air samples were collected in several areas around Shaoguan city, including indoor market places (three supermarkets and one bookstore), one playground and one hospital. The number of sampling points (from four to eight points) depends on the size of each sample place. 67 air samples collection was determined by the atmospheric articles PM10 and PM2.5 in atmosphere via gravimetric method mentioned in Ministry of Environmental Protection of the People’s Republic of China (HJ 618–2011)^29^. Air samples were collected by an atmospheric dust sampler running for 20 minutes at a speed of 20 L/min. Another 92 samples were collected via wiping entertainment facilities in ten different children playgrounds in Guangzhou city. All samples were placed into viral transport medium (VTM) immediately after collecting, and sent back to the laboratory, stored at −80 °C temperature refrigerators. VTM was self-configured as with 0.4 g potassium chloride, 6.8 gsodium chloride, 2.2 g sodium bicarbonate, 0.14 g sodium dihydrogen phosphate monohydrate (NaH_2_PO4·H_2_O), 1 g D-glucose, 300 μl Amikacin sulphate (0.1 g/ml), 100 mg vancomycin hydrochloride, 8 mg nystatin and 50 g bovine serum albumin, as water was added to 1000 ml graduated measuring cylinder together. VTM was filtered and sterilized.

### RNA extraction

All the samples were taken out of the refrigerators, defrosted within ice environment and homogenized by Vortex oscillation apparatus. 200 μl solution of each samples were processed by using the QIAamp MinElute Virus Spin Kit (50 reactions) (QIAGEN, Germany) according to the manufacturer’s instructions. RNA was extracted and stored under −80 °C temperature.

### Reverse transcription and real-time fluorescence quantitative PCR

Extracted RNA was detected by reverse transcription and qPCR. According to standardized protocols from Chinese Centre For Disease Control And Prevention (Hand-Foot-Mouth Disease Control and Prevention Guide, 2009 Edition)^30^, extracted RNA was taken from the refrigerators and defrosted with ice. The reverse transcription kit in this study was Transcriptor First Strand cDNA Synthesis Kit (200 reactions) (Roche, Germany). Sample cDNA that generated from the reverse transcription was stored under −80 °C temperature. Referring from previous studies in literature review^27^,^31^,^32^, primers were designed (Invitrogen Custom Primers, shanghai). Details of the primers were listed in Table 3. 1 ul probe, 1 ul upstream primer and 1 ul downstream primer were added with10 ul qPCR master mix (LightCycler 480 Probes Master, Roche, Germany) into each tube. 7 ul sample cDNA was added into each tube, qPCR proceeded with quantitative fluorescence analyser (Bio-rad CFX96, America). When Ct value was less than 40 or greater than 20, corresponding children’s caregiver samples would be considered as having positive results.

### Statistical analyses

Data was analysed by SAS statistical software (version 6.12; SAS Institute, Cary, NC). Pearson Chi-square test or Fisher’s exact test were used for categorical data in this study. When any expected frequency was less than 5, Fisher’s exact test was chosen for analysis, otherwise Pearson Chi-square test would be applied. The alpha level for all tests was 0.05.

## Additional Information

**How to cite this article**: Pengyuan, L. *et al*. Children’s Caregivers and Public Playgrounds: Potential Reservoirs of Infection of Hand-foot-and-mouth Disease. *Sci. Rep.* **6**, 36375; doi: 10.1038/srep36375 (2016).

**Publisher’s note:** Springer Nature remains neutral with regard to jurisdictional claims in published maps and institutional affiliations.

## Figures and Tables

**Table 1:** Viruses detection amounts via real-time fluorescence quantitative PCR.

Sample name	Sample type	Number	Detection results			
			UEV+^1^	EV71+^2^	CVA16+^3^	CVA6+^4^
**Children’s caregiver sample**	**Nasal and throat swab**	**98**	**7 (7.14%**, **95% CI**^5^**: 2.04%**, **12.24%)**	**0**	**0**	**0**
**Environmental sample**	**Air/wiping facilities**	**159**	**8 (5.03%**, **95% CI: 1.63%**, **8.43%)**	**0**	**0**	**0**
Playgrounds in Guangzhou	Wiping facilities	92	7 (7.61%, 95% CI: 2.21%, 13.01%)	0	0	0
Public area in Shaoguan	Air	67	1 (1.49%, 95% CI: 0, 7.00%)	0	0	0
**Total**	**—**	**257**	**15 (5.84%**, **95% CI: 2.98%**, **8.70%)**	**0**	**0**	**0**

^1^UEV+: Universal enterovirus positive.

^2^EV71+: Enterovirus 71 positive.

^3^CVA16+: Coxsackievirus A16 positive.

^4^CVA6+: Coxsackievirus A6 positive.

^5^95% CI: 95% confidence interval.

**Table 2:** Children caregiver samples in healthy adults or with HFMD recessive infections in Guangzhou.

Age	Number of samples	Number of UEV+^1^ samples	Positive rate (95% confidence interval)
20–39	64	4	6.25% (0.32%, 12.18%)
>=50	26	3	11.54% (2.00%, 30.00%)
Unknown	8	0	0
Total	98	7	7.14% (2.04%, 12.24%)

^1^UEV+: Universal Enterovirus positive.

**Table 3:** Primers for real-time fluorescence quantitative PCR.

Virus Name	Primer Name	Sequence 5′–3′*	3′ Label	5′ Label	Size (bp)	Gene Target
**Enterovirus** ^**36**^	EV-Forward primer	CCCTGAATGCGGCTAATCC				
	EV-Reverse primer	ATTGTCACCATAAGCAGCCA				
	EV-Probe	AACCGACTACTTTGGGTGTCCGTGTTTC	BHQ1	FAM	146	Polyprotein
**CV-A16** ^**37**^	CV-A16-Forward primer	CCTACAGCTGCCAACACTGAGG				
	CV-A16-Reverse primer	CATTAGACGACGCCCCTGTCTC				
	CV-A16-Probe	CAYAGATTAGGCACTGGTGTTGTACCAGCA	BHQ1	FAM	94	VP1
**EV71** ^**37**^	EV71 -Forward primer	TTCATGTCACCYGCGAGYGC				
	EV71-Reverse primer	GCYCCRTATTCAAGRTCTTTCTC				
	EV71-Probe	TAYGACGGRTAYCCCACRTTYGGWGA	BHQ1	FAM	92	VP1
**CV-A6** ^**38**^	CV-A6-Forward primer	CAAGCTGCAGAAACGGGAG				
	CV-A6-Reverse primer	GCTCCACACTCGCCTCATT				
	CV-A6-Probe	ACCCCGTTTCGATTCATCACACA	BHQ1	FAM	103	VP1

^*^R = A or G; Y = C or T; W = A or T.
